# Thinking big: towards ideal strains and processes for large‐scale aerobic biofuels production

**DOI:** 10.1111/1751-7915.12471

**Published:** 2016-12-22

**Authors:** James D. McMillan, Gregg T. Beckham

**Affiliations:** ^1^ National Bioenergy CenterNational Renewable Energy Laboratory15013 Denver West ParkwayGoldenCO80401USA

Global concerns about anthropogenic climate change, energy security and independence, and environmental consequences of continued fossil fuel exploitation are driving significant public and private sector interest and financing to hasten development and deployment of processes to produce renewable fuels, as well as bio‐based chemicals and materials, towards scales commensurate with current fossil fuel‐based production. Over the past two decades, anaerobic microbial production of ethanol from first‐generation hexose sugars derived primarily from sugarcane and starch has reached significant market share worldwide, with fermentation bioreactor sizes often exceeding the million litre scale. More recently, industrial‐scale lignocellulosic ethanol plants are emerging that produce ethanol from pentose and hexose sugars using genetically engineered microbes and bioreactor scales similar to first‐generation biorefineries.

Concomitant with the genesis of a lignocellulosic fuel ethanol industry, publicly funded research by the academic and governmental scientific community has largely shifted its research and development emphasis to producing biofuels that can be drop‐in replacements for fossil‐based diesel, gasoline and jet fuels. This research pivot reflects both limitations in infrastructure compatibility and associated (real or perceived) concerns with ethanol as a fuel blendstock, as well as growing promise of new capabilities in microbial genetic modification emerging through advances in metabolic engineering and synthetic biology. For biofuels produced primarily via microbes, most proposed target drop‐in fuels or fuel precursors are or will be produced via pathways ‘deep’ in carbon metabolism, such as fatty acid, isoprenoid or polyketide synthesis pathways (Peralta‐Yahya *et al*., 2012). Myriad perspectives, reviews and original research reports have been published regarding optimal metabolic engineering, systems biology, and synthetic biology strategies for producing drop‐in biofuels or fuel precursors using these pathways. Undoubtedly, this overarching strategy to redirect central carbon metabolism exhibits great promise for developing strains capable of producing a range of biofuels suitable for multiple transportation markets and for producing a wide variety of commodity and fine chemicals.

While a wide range of drop‐in biofuels and bio‐based products can be produced via the aforementioned metabolic pathways, these pathways are as yet only known to be highly productive under aerobic conditions. This is because these synthesis pathways to produce hydrocarbon or near‐hydrocarbon products (e.g. fatty acids and long‐chain alkanes) are thermodynamically ‘uphill’ anabolic metabolic pathways that require energy in the form of ATP and NAD(P)H to achieve high production rates, i.e. as opposed to the thermodynamically ‘downhill’ catabolic metabolic pathways used to produce ethanol (or butanol or short‐chain carboxylic acids) by anaerobic fermentation, which are net ATP‐positive, redox balanced, and can be performed anaerobically.

The use of metabolic pathways requiring aerobic respiration to generate the energy needed to drive them, while reasonable for producing high‐value fine chemicals, therapeutics, amino acids, antibiotics, and even some higher value commodity chemicals, poses serious challenges for economic production of huge‐volume, lower cost fuels. This is primarily due to the additional capital and operating costs required to supply oxygen (O_2_) to a submerged culture and secondarily due to reduced economies of scale for scaling up aerobic versus anaerobic processes. As an example, the higher costs for aerobic versus anaerobic production are illustrated by comparing two recent NREL design case reports based on largely similar process designs: much higher costs are projected to aerobically produce renewable diesel blendstock using oleaginous yeast (Biddy *et al*., 2016), than to anaerobically produce ethanol (Humbird *et al*., 2011). As such, for a given target pathway and product, key questions related to oxygen requirements and production host robustness need to be addressed early in the process development cycle, ideally during initial strain selection and metabolic engineering phases, questions that generally have not needed to be asked previously (i.e. for anaerobic production). While oxygen transfer and scale up are well understood and developed for many bio‐based products, such as amino acids and antibiotics, biofuels represent a larger scale, lower margin opportunity for which there still exist many questions and unknowns. Foremost, for biofuels (and other commodity bio‐based products), it remains unclear how large and how low cost aerobic production can be.

This Crystal Ball perspective proposes several strain attributes and strain selection criteria that will be highly beneficial to consider *a priori* to effectively develop engineered microbes well suited for large‐scale aerobic cultivation to produce biofuels or biofuel precursors. We posit that these points should be seriously considered by metabolic engineers and synthetic biologists trying to develop industrially relevant microbes fit for large‐scale aerobic production of biofuels and commodity bioproducts. Maximizing strain robustness will be essential to enable larger scale aerobic production than has been demonstrated to date and thus be a key to overcoming the grand‐challenge science and engineering problem of cost‐competitively using renewable biomass to displace fossil fuels, while simultaneously boosting the bioeconomy and providing a path to a more sustainable future.

Thinking big about what attributes biofuels production strains will need benefits from envisioning at the outset what large‐scale production will look like and thoroughly considering how large‐scale conditions will differ from those typically encountered in smaller scale laboratory bioreactors (Blanch, 2012; Davison and Lievense, 2016). First, appreciable vessel mixing times and associated gradients in temperature, hydrostatic pressure, nutrient concentrations and dissolved O_2_ (and perhaps dissolved CO_2_) concentration, which are not typically significant at bench scale, will be present in full‐scale bioreactors. Ultimately, new process designs may be required to achieve sufficient mixing in the bioreactor vessel so that under proper operation, the magnitude and duration of such gradients will remain within what the production strain can accommodate. Achieving favourable process economics requires extreme cost minimization, however, and will likely include minimizing energy inputs for mixing and aerating the culture, i.e. power for agitation and air/oxygen compression, and lowering these energy inputs will generally translate into larger gradients within the bioreactor. As such, beyond identifying native or engineered strains that possess desired or more desirable metabolic pathways, strain selection needs to place greater focus on identifying production strains capable of maintaining robust performance over a wider range of temperatures and nutrient concentrations than are typically being studied and reported on in recent literature. Simply put, for extremely large‐scale production, strains will be favoured that can remain productive in the presence of larger concentration and temperature gradients within the bioreactor over the course of the production process.

For robust cultivation on lignocellulosic sugars, there are several general, well‐known strain selection criteria. Foremost are the ability to achieve high cell mass growth and biofuels production rates in biomass‐derived hydrolysates, which often contain a variety of inhibitory components, including aromatic compounds derived from lignin, acetate from hemicellulose, and aldehydes from sugar dehydration reactions. Additionally, the ability to utilize a broad range of pentose and hexose sugars is tremendously beneficial, with the caveat that if needed the ability to utilize additional sugars likely can be engineered into a strain, although this is often a non‐trivial undertaking. Lastly, the ability to tolerate high temperatures or low pH (or lack of pH control) is often a key driver in strain selection. This is because operating a bioreactor at higher temperature generally makes temperature control easier and more economical. Higher temperature also favours higher reaction rates, lower cultivation broth viscosities, and in some cases can also be useful to reduce contamination risk during production. Similarly, the ability to tolerate lower pH can be useful to reduce contamination by many bacteria.

In general, the case can be made that, to date, much of the effort to develop strains for large‐scale biofuels production has been focused on what is known and can be carried out today rather than on what needs to be carried out to achieve the unprecedented levels of process efficiency and cost minimization that will be required to economically produce relatively low priced and low margin biofuels in submerged aerobic bioprocesses. The literature shows recent work being carried out predominantly using well‐characterized microbes and idealized experimental conditions we are most familiar with to demonstrate ‘proof of concept’ of new proposed production routes rather than identifying strains that are sufficiently robust to perform well in envisioned large‐scale processing conditions where higher concentrations and larger concentration and temperate gradients will be present. This is exemplified by the predominance of the use of well‐known microbes like *Escherichia coli*, which is advantageous for being genetically tractable and reasonably well understood, but also has many undesirable traits such as relatively low tolerance to acids and low pH, no well‐established coproduct value for its cell mass, and limited thermotolerance. It is also reflected by a dearth of new public domain literature on larger scale bio‐based aerobic production over the past 20+ years. Notably, the literature on oxygen transfer and aeration for submerged production published over the last several decades is dominated by reports of mammalian and microbial cell‐based pharmaceutical/therapeutic production, with bioreactor of volumes in the range of 20 000 L deemed ‘large scale’; this is orders of magnitude smaller than the 1 000 000 L and larger scales considered relevant for submerged aerobic fuel production.

In addition to the well‐known titre, rate and yield (TRY) performance criteria, another strain/process attribute that can make or break the viability of a large‐scale production process is broth rheology, which if too high can hinder mass transfer (e.g. O_2_ gas‐liquid mass transfer) and heat transfer as well as constrain the size to which the process can be economically scaled. For this reason, research on aerobic production has generally used clarified (solid‐free) sugars as a carbon source, i.e. rather than whole slurry hydrolysates as generated by enzymatic hydrolysis of pretreated biomass. Regardless, it is essential that to maintain a sufficiently low broth viscosity that good mixing and O_2_ transfer can occur, and it is important to minimize the presence of non‐host microbe solids. Similarly, to maintain effective mixing and aeration, a production strain must not form appreciable levels of any coproducts such as exopolysaccharides (EPS) that will significantly increase the viscosity of the culture broth. In general, feedstock carbon should not be directed to non‐target products in any manner that lowers product yield.

Finally, considering the overall production process, a highly desirable strain attribute is to be able to secrete the fuel or fuel precursor product into the culture broth, or if produced as an intracellular product, to be able to easily rupture the product‐laden cells to obtain the product. This is because the cost of product recovery and purification can be substantial, and if an energy‐intensive cell rupture step is required, such costs may become prohibitive for producing a biofuel (Biddy *et al*., 2016).

Looking forward, another more complex but powerful strategy with growing potential given the synthetic biology pathway engineering tools now available is to re‐engineer biofuels (or biofuels precursors) production metabolic pathways to increase their energy efficiency and thereby reduce their oxygen demand and related aeration energy requirements. A leading example of successfully applying this approach was recently reported by Amyris (Meadows *et al*., 2016), wherein synthetic biology techniques were used to ‘rewire’ central carbon metabolism in *Saccharomyces cerevisiae* to increase the efficiency of acetyl‐CoA‐based isoprenoid production, with the modified pathway impressively achieving 25% higher product yield on sugar while requiring 75% less oxygen for aeration.

Finally, microbial electrosynthesis (MES) technology is emerging as an intriguing possibility for enhancing bioprocess efficiency by enabling renewable energy in the form of green electrons to supply some of the reducing power needed to drive anabolic product synthesis (Schievano *et al*., 2016). MES exploits the observed but not yet well‐understood phenomenon of extracellular electron transfer between electrogenic microbes and solid electrodes. Whether such an approach can be used to improve aerobic production remains an open question. MES is still at an early stage of development, so far only demonstrated for a few anaerobic strains (e.g. acetogens) and processes and much research and development remains to be carried out to establish economically compelling routes incorporating MES. Nonetheless, it will be highly advantageous to be able to drive aerobic microbial production using green electrons produced by wind, geothermal, solar, or other renewably sourced power systems, rather than having to oxidize a significant fraction of the sugar carbon source substrate to generate reducing power at the expense of reduced product yield.
